# Seizures and Sepsis: A Narrative Review

**DOI:** 10.3390/jcm10051041

**Published:** 2021-03-03

**Authors:** Francesco Alessandri, Rafael Badenes, Federico Bilotta

**Affiliations:** 1 Department of Anesthesia and Intensive Care Medicine, “Sapienza” University of Rome, Policlinico Umberto I, 00161 Rome, Italy; F.Alessandri@policlinicoumberto1.it (F.A.); bilotta@tiscali.it (F.B.); 2 Department Anesthesiology and Surgical-Trauma Intensive Care, Hospital Clinic Universitary, 46010 Valencia, Spain; 3 Department of Surgery, University of Valencia, 46010 Valencia, Spain

**Keywords:** seizures, septic encephalopathy, cEEG, anticonvulsants

## Abstract

Patients with sepsis-associated encephalopathy (SAE) can develop convulsive or nonconvulsive seizures. The cytokine storm and the overwhelming systemic inflammation trigger the electric circuits that promote seizures. Several neurologic symptoms, associated with this disease, range from mild consciousness impairment to coma. Focal or generalized convulsive seizures are frequent in sepsis, although nonconvulsive seizures (NCS) are often misdiagnosed and prevalent in SAE. In order to map the trigger zone in all patients that present focal or generalized seizures and also to detect NCS, EEG is indicated but continuous EEG (cEEG) is not very widespread; timing, duration, and efficacy of this tool are still unknown. The long-term risk of seizures in survivors is increased. The typical stepwise approach of seizures management begins with benzodiazepines and follows with anticonvulsants up to anesthetic drugs such as propofol or thiopental, which are able to induce burst suppression and interrupt the pathological electrical circuits. This narrative review discusses pathophysiology, clinical presentation, diagnosis and treatment of seizures in sepsis.

## 1. Introduction

Sepsis is a life-threatening condition due to dysregulation of the body’s response to infection that induces damage to its own tissues and organs [1]. Sepsis-associated encephalopathy (SAE) complicates the course of up to 70% of septic patients and can present various clinical pictures: inattention, confusion, delirium, excitation, seizures, stupor and coma [2,3]. Up to 20% of critically ill patients develop seizures, which can present as convulsive (focal or generalized) or nonconvulsive (NCS); the latter represent 90% of the cases [4]. After an episode of generalized convulsive status epilepticus, occurrence of NCS is diagnosed in 48% of patients [3]. The delay in diagnosis or treatment of nonconvulsive status epilepticus (NCSE) in SAE patients associates with an increase in morbidity and mortality [5]. Furthermore, in septic patients who survive and are discharged home, the risk for long-term consequences of seizures is higher than in the general population and in other hospitalized patients, suggesting that sepsis associates with permanent neurologic sequelae [6]. Several aspects of SAE have been described that include cognitive dysfunction predominantly affecting general memory, attention, verbal fluency, delirium [7]. A thorough analysis of immediate (including diagnostic indications and therapeutic options) and long-term (prognosis and prevention) implications of seizures in septic patients is lacking.

The aim of this narrative review is to report on pathophysiology, clinical presentation, diagnosis, treatment and long-term implications of seizures in patients with sepsis.

## 2. Methods

A PubMed database search was formulated using the following search criteria: seizures AND sepsis, SAE AND seizures, Electroencephalography AND sepsis. Filters: Humans; English; Adult: 19+ years. Articles published in the English language, in indexed scientific journals, were included. Only studies performed in adults were eligible for inclusion. The research was updated on December 2020. The references for all included papers, review articles, and commentaries, and editorials on this topic were also reviewed to identify other studies of interest that were missed during the primary search. We did not perform a systematic review.

## 3. Pathophysiology

Sepsis is characterized by a tight interaction between the infection and the host response that leads to an overwhelming systemic inflammatory release and cytokines storm that exert excitatory effects in the central nervous system through various mechanisms that include: N-methyl-D-aspartate (NMDA), γ-amino-butyric-acid (GABA) and increased vascular permeability [8,9]. Furthermore, the combined actions of proinflammatory and anti-inflammatory factors play a role in the neuropathology of acute brain dysfunction and sepsis-related seizures [9]. An increased level of circulating cytokines (as IL-1β, TNF-α, IL-6, IL-12) and migration inhibitory factor (MIF) of macrophage, documented in central nervous system inflammation, are proved to be involved in the neuronal excitotoxicity pathways [9]. In particular, the systemic increase in IL-1β concentration induces the activation of the neuronal IL-1R1 and the subsequent phosphorylation of NR2B subunit of NMDA receptor, which turn into NMDA-mediated calcium influx into neurons [10]. The IL-1β also interferes with GABA-mediated inhibitory synaptic transmission [11]. Another recognized cause of seizure is the increase in extracellular concentration of glutamate: TNF-α, released from glia, increases this crucial neurotransmitter, while other cytokines decrease its reuptake by astrocytes [9,12]. The increase in vascular permeability of the blood-brain barrier (BBB) promoted by nitric oxide and tight junction damage enhance neuronal environment hyperexcitability [13]. In septic patients, the enhanced permeability of BBB increases the translocation of cytokines, carrying the proinflammatory response from the serum into the brain and favoring seizure susceptibility. The damage of BBB could induce seizures as well as in post-traumatic epilepsy [9,14].

## 4. Clinical Presentation

Patients affected by SAE can present various neurologic symptoms, which range from mild consciousness impairment to coma; of note, none of the symptoms or signs specifically and selectively predict seizures. Both focal (i.e., nystagmus, eye flutter, blinking, and eye deviation) generalized signs as myoclonus tremulousness and autonomic instability may be present. Motor findings are not constant or necessarily present, and in some cases, EEG is needed for the differential diagnosis with myoclonic jerks associated with electrographic discharges from nonepileptic myoclonus that are frequent in other kinds of toxic or metabolic encephalopathy [15,16]. Neglect syndrome, apraxia, aphasia, amnesia, homonymous hemianopia, and hemiparesis, are rarely associated with seizures, and are often confused with stroke without focal imaging on TC scan [17]. In elderly patients, clinical presentation of seizures can be extremely atypical: paroxysmal nonepileptic events, inattention, memory lapses, confusion and prolonged postictal state can be misdiagnosed and attributed to “senior moments” or early signs of dementia. Seizures in elderly are longer in duration and associated with a higher mortality risk, particularly when are treated with benzodiazepines [18,19].

Seizures can occur in patients without history of epilepsy or induced by several factors such as sleep deprivation, noncompliance with antiepileptic medications, fever or stress [20]. Seizures can last for a few hours to several days. A full EEG is necessary to confirm the diagnosis [21].

## 5. Electroencephalography

Electroencephalography (EEG) is a relatively simple, cheap tool for the noninvasive monitoring of brain activity that has a pivotal role in ICU patients [22]. It is a cornerstone of the diagnostic work-up in patients with consciousness disorders because of its ability to detect structural and functional patterns of neuronal activity [23]. The use of 21-leads EEG is indicated in all patients that present focal or generalized seizures for differential diagnostic purposes, to identify the specific electrical profile and to map the trigger zone involved areas [24]. Despite the accuracy of information provided by the EEG, it is important to note that sometimes this exam can be misleading due to concurrent treatment with sedatives, antipsychotics, antiseizure drugs, opiates and antibiotics (i.e., penicillin and cephalosporin). These drugs are frequently used in critically ill patients and can induce a “shadowing effect” [25].

**Figure 1:**
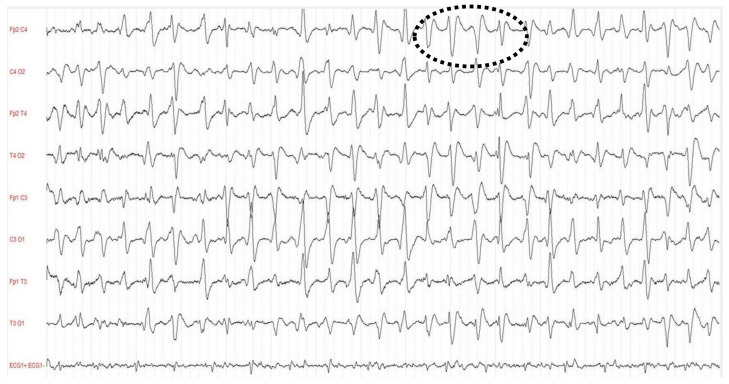
Triphasic wave in an electroencephalography (EEG) of patient with sepsis-associated encephalopathy (SAE).

Evidence that supports the role of EEG in SAE is sparse [4,26]. The EEG-derived electric patterns reported in septic patients are characterized by a progressive slowing correlated with the level of consciousness, and these include: mild slowing of theta waves, severe slowing of delta waves, periodic discharges (PDs), generalized periodic discharges (GPDs), GPDs with triphasic morphology (Figure 1), electrographic seizures (ESZs), generalized suppression, or even burst suppression [27,28]. In adult patients with SAE, the incidence of EEG abnormalities ranges from 12 to 100% for background abnormality and 6 to 12% for presence of triphasic waves [29]. The latter are characteristically detected in patients presenting hepatic failure or sepsis both when associated or not with multiorgan failure. In a single center retrospective study that reported data on 201 consecutive patients admitted to ICU without known acute neurologic injury and who underwent cEEG for investigation of possible seizures or changes in mental status, ESZs or PDs were more common in septic critically ill patients than in those without sepsis (32% vs. 9%) [30]. There is a relationship between the severity of EEG abnormalities and presence of SAE [30,31]. However, the interpretation of EEG in critically ill septic patients is often compromised by the lack of clear clinical definition for sepsis-associated brain dysfunction. The ICU patient’s mortality is linearly associated with severity of EEG abnormalities and reaches 36% in patients that present delta waves, 50% in those with triphasic waves, and 67% when EEG shows burst suppression. In a recent prospective study that investigated the prevalence, risk factors and impact of continuous electroencephalogram (cEEG) abnormalities in 98 ICU septic patients, the lack of EEG reactivity was associated with increased mortality up to one year after ICU discharge, while NCS and PDs were common in patients with severe sepsis and altered mental status but were not associated with worse outcomes [32]. However, PDs lasting longer than 24 h have been identified as independent predictors of poor outcome. Early standard short-duration EEG trial also demonstrated the link between lack of EEG reactivity and increased mortality. In septic patients with unexplained mental status impairment, cEEG monitoring is recommended [4]. When PDs last longer than 24 h they are independent predictors of poor outcomes. Relevance of cEEG was confirmed in an observational study of 200 medical ICU patients—60% septic and 48% comatose—monitored to investigate the incidence of seizures that were detected in 67% of the cases without clear clinical correlate [33]. The duration of cEEG necessary to detect NCS is unknown and there are no available dedicated studies that compared continuous to intermittent EEG. In a large retrospective study of 625 monitored ICU patients, the early presence of EEG abnormalities accurately stratified the risk of seizures within 72 h: decreasing below 10% and 5% at 7 and 16 h, respectively, after the appearance of epileptiform discharges; on the contrary, in patients without epileptiform abnormalities, the 72-h seizure probability dropped below 10% at 15 min and to 5% at 2 h [34]. On the other hand, intermittent EEG was shown to insufficiently detect NCS: in a prospective observational cohort study of 110 patients with sepsis an early standard short-duration EEG did not provide an accurate prevalence of EEG abnormalities in septic patients (periodic discharges and electrographic seizures were found in about 20% of patients). However, the authors concluded that the prevalence of EEG abnormalities could have been higher, with a still higher predictive value, if they had been diagnosed using cEEG [35,36]. Considering that 80–95% of NCS can be identified within 24–48 h, a cEEG run in all septic ICU patients is recommended to accomplish at least 24 h, making this monitoring approach a possible standard of care in clinical management [37,38]. The use of cEEG monitoring should be initiated as soon as possible when NCS are suspected [39].

## 6. Seizures and Long-Term Complications

In patients that develop seizures during sepsis there is a relevant risk of long-term seizure recurrence [6]. As reported in a large retrospective data analysis of septic emergency department patients, long-term risk of seizures in survivors is higher than in other hospitalized patients and 5 times higher than in the general population [6]. Several confounding factors such as preexisting cognitive impairment, frailty, prolonged effects of sedative drugs, and others prevent the assessment of long-term cognitive dysfunction after sepsis [40,41,42]. Different mechanisms are involved in the pathophysiology of long-term cerebral effects after seizures: brain inflammation, disruption of the blood-brain barrier, cerebral blood flow impairment, metabolism and mitochondrial dysfunction, cellular apoptosis and inhibition of neurovascular coupling [43,44,45]. Apart from neurologic impairment, the specific risk factors for chronic epilepsy that could lead to defining the optimal management of sepsis survivors —including the need and frequency of EEG monitoring, need and duration of seizure prophylaxis therapy—are still unknown. Given these premises, in ICU patients it is necessary to pay the highest attention to early diagnosis of seizures and to start effective therapy to prevent acute and long-term complications of undetected seizures [46].

## 7. Treatment

Prevention and treatment of seizures in sepsis have been poorly addressed. Available evidence suggests that is essential to quickly control any seizure activity in both clinically evident status epilepticus and NCS detected on cEEG (Table 1) [47].

The typical stepwise approach of seizure management begins with a prompt administration of benzodiazepines [48]. Among these, midazolam and lorazepam are preferred due to the lower distribution volume and the lower seizure recurrence risk than diazepam [49]. These drugs work by increasing the activity of the GABA neurotransmitter in the brain. The onset time of midazolam is 5 min after intravenous dose or 15 min after intramuscular dose; its effect lasts from 1 to 6 h. Intravenous lorazepam typically begins working within 1–5 min and its effect lasts for 8–12 h because of a favorable distribution coefficient allowing lorazepam to remain in the brain longer than other benzodiazepines. Respiratory depression, hypotension and sleepiness are typical side effects of benzodiazepine, especially after intravenous administration [48].

The second line treatment should be considered to stop seizures in those patients still seizing despite benzodiazepine therapy, or to prevent recurrent seizures in those patients treated successfully with benzodiazepines and includes the use of longer-acting anticonvulsants such as phenytoin, valproic acid, and levetiracetam [50]. Phenytoin is an Na+ channel blockade, with an onset time of 10–30 min and a duration of action of about 24 h. Phenytoin is not soluble in water; therefore, its emulsion can cause tissue necrosis in case of extravasation. Fosphenytoin is the hydrolyzed prodrug of phenytoin, but the need for plasma conversion to the active drug results in a comparable time to peak plasma levels when compared with phenytoin administration itself. Adverse effects include hypotension and arrhythmias [51]. Valproic acid acts on GABA levels in the brain, blocks voltage-gated ion channels, and also acts as a histone deacetylase (HDAC) inhibitor [52]. The rapid onset of the anticonvulsant action after intravenous bolus injection of valproate is related to rapid drug penetration into brain tissue; this drug suppresses seizures also at nonsedative doses [52]. Concentration-related side effects include ataxia, lethargy, tremor, and coma [51]. Dizziness, thrombocytopenia, and mild hypotension are adverse events independent of the infusion rate. Acute encephalopathy is sometimes related to hepatic abnormalities or hyperammonemia. Valproic acid dose is poorly related to total serum concentrations, due to significant interpatient differences in valproic acid metabolism and its high protein binding [52]. Levetiracetam is an attractive therapeutic choice for treatment of seizures in ICU. It is a small hydrophilic molecule with little protein binding [53]. This drug decreases calcium-dependent vesicular neurotransmitter release binding to the synaptic vesicle protein 2A (SV2A), which is part of secretory vesicle membranes [53]. Other mechanisms include inhibition of α-amino-3-hydroxy-5-methyl-4-isoxazolepropionic acid (AMPA) receptor and modulation of calcium channels and of GABA and glycine neurotransmission [54]. Lacosamide, the R-enantiomer of 2-acetamido-N-benzyl-3-methoxypropionamide, selectively enhances the slow inactivation of voltage-gated sodium channels and increases neuronal depolarization thresholds [55]. It reduces seizure frequency in patients with uncontrolled partial onset seizures [55]. Lacosamide can be considered an alternative to fosphenytoin in the treatment of NCSs detected on cEEG [56].

In patients with refractory status epilepticus, and when patients are intubated and ventilated, the use of anesthetic drugs such as propofol, thiopenthal or large doses of midazolam is required to induce coma in order to switch off the electric circuits and to achieve complete control of seizures [50]. Thiopental sodium, a barbiturate, is a GABA-A agonist with a prolonged duration of action, mainly due to accumulation in the body [54]. Propofol has a uniform depressant action on the central nervous system, to a potentiation of GABA-A mediated pre- and postsynaptic inhibition by enhancing inward GABA-A Cl currents, and by decreasing the release of excitatory transmitters glutamate and aspartate [57]. Propofol has a shorter duration of action and better pharmacokinetic than thiopental. Despite anesthetic drugs reducing intracranial pressure and brain metabolic requirements, especially in septic patients, these can lead to several systemic dysfunctions such as hypotension and respiratory depression [58]. Anesthetic drug administration has to be monitored with cEEG in order to achieve either seizure cessation or background suppression [59]. Despite burst suppression allowing for the brain to rest and recover, the disadvantage might be a worse outcome associated with prolonged intubation and hospitalization [60]. In most cases, a loading dose of an intravenously antiepileptic drug is administered, but often a combination of drugs is required: drugs’ interaction may reduce the antiepileptic drug concentration and the seizure threshold [61]. Antibiotics, psychotropic agents, and analgesics have been all associated with seizures: most of them can interfere with metabolism and influence pharmacokinetics of antiepileptic drugs [62]. There is no evidence that supports the prophylactic use of anticonvulsive therapies [63].

## 8. Discussion

This narrative review describes clinical evidence on seizures in patients with sepsis. This serious complication is often underdiagnosed despite the fact that it can significantly contribute to worsening the clinical picture and the outcome of these patients. Monitoring brain activity in septic patients with elective EEG monitoring is indicated, and cEEG is mandatory in selected patients not only to verify the efficacy of anticonvulsant therapy but also to identify NCS. Because of the unique background pathophysiology, the treatment of seizures in septic patients benefits from a specific therapeutic approach and can aid the improvement of the inflammatory response to the infective status.

Sepsis is a syndrome with a vast array of causes, effects and different underlying mechanisms. However, there are common mechanisms of seizure generation and propagation that can be promptly treated. Otherwise, systemic manifestations of status epilepticus will complicate a patient’s ICU course. In the setting of convulsive status epilepticus, patients can develop a metabolic acidosis secondary to muscle contraction leading to anaerobic metabolism with lactic acid formation; this can lead to a poor prognosis in these critical septic patients. In acute brain-injured patients, seizures complicate the clinical course with an incidence as high as 5 to 15%, and this is possibly due to the cerebral release of excitatory amino acids, neurotransmitters and hyperglycolysis [64,65]. Some present with NCS while others show symptoms that range from blurred (i.e., oral or ocular movements and/or gaze deviation) to open crisis [66]. In patients with intracranial hemorrhage, seizures are more frequent than in brain ischemic injury, probably because extravasated iron is potentially proconvulsive [67]. The sensitivity of intermittent EEG to diagnose NCS in comatose patients is poor when compared to a sensitivity on a 48-h cEEG [64]. Use of cEEG monitoring in ICU is limited due to high cost, nonavailability of technicians to apply and maintain electrodes, considerable ICU-related EEG artifacts, and availability of physicians for a timely interpretation of the EEG. Devices for automated seizure detection and remote access for EEG viewing are being developed to overcome these limitations [68].

This narrative review has several limitations: First, the context is limited to septic ICU patients, and therefore excluded other clinical settings (i.e., outpatients and non-ICU patients). Second, few studies describe the best diagnostic approach and no information is available on specific risk factors of seizures in this cohort of patients affected by sepsis. And last, no observational prospective studies are available on the optimal follow-up of this brain disarrangement cause of seizures in septic patients.

## 9. Conclusions

In conclusion, current understanding of the mechanisms underlying the pathogenesis of seizure is of paramount interest for researchers to improve the early diagnosis of seizures by using cEEG recording. The ability to predict seizures prior to onset could transform the current mode of therapy for administering of medication well before the seizure occurs. Collaborative efforts and prospective studies are needed to improve the management of seizures in patients affected by SAE.

## Figures and Tables

**Table 1:** Pharmacology of anticonvulsant drugs.

	I.V. Dose	Onset Time Duration	Protein Binding Half Life	Metabolism and Elimination	Complications
Lacosamide [55,56]	100–200 mg/day	1–4 h (time to peak)	<15% T_1/2_ = 13 h	Hepatic via CYP2C19, 40% unchanged drug 95% renal	dizziness, nausea, blurred vision, and imbalance, atrial flutter, PQ interval prolongation and AV-block)
Levetiracetam [50]	3000 mg or 60 mg/kg 2–5 mg/kg/min	5–30 min	<10% T_1/2_ = 6–8 h	Non-hepatic hydrolysis ~66% renal as unchanged drug	Agitation, irritability, psychotic symptoms
Lorazepam [48,49]	0.1-mg/kg	1–5 min 12–24 h	~91% 12–18 h	Hepatic; rapidly conjugated to inactive metabolite ~88% renal as inactive metabolites	Respiratory depression, Hypotension
Midazolam [48,49]	0.02–0.3 mg/kg 0.05–0.1 mg/Kg/h	2 min 1–6 h	~97% T_1/2_ = 3 h	Extensively hepatic CYP3A4; 60% to 70%to active metabolite ~90% renal as metabolites	Respiratory depression, hypotension
Phenytoin [51]	PE 20 mg/kg at 150 mg/kg/min PE 20 mg/kg at 50 mg/min	10–30 min 24 h	90–95% T_1/2_ = 7–42 h	Fos: Prodrug, rapidly hydrolyzed to phenytoin. Hepatic via CYP2C9, 2C19, 3A4 <5% renal as Phenytoin metabolites	Hypotension, phlebitis, cardiac arrhythmias.
Propofol [57]	1–2 mg/kg 20–80 mcg/kg/min	15–30 s	97–99% T_1/2_ = 40 min ^§^	Hepatic to water-soluble sulfate and glucuronide conjugates ~90% renal as metabolites	Respiratory depression hypotension, PRIS
Thiopental [54]	3–5 mg/Kg 2–10 mg/Kg/h	30–40 s 5–10 min	80% T_1/2_ = 12 h	Hepatic metabolized to pentobarbital Renal	Respiratory depression
Valproic Acid [52]	20–40 mg/kg 3–6 mg/Kg/min	30 s	80–90% T_1/2_ = 9–19 h	Hepatic via glucuronide conjugation and mitochondrial beta-oxidation 50–80% renal	Hepatotoxicity, pancreatitis, thrombocytopenia, hyperammonemia

AV-block = Atrio-ventricular block; PE = phenytoin equivalents; PRIS = propofol-related infusion syndrome; FOS = Fosphenytoin; T_1/2_ = half-life. ^§^: prolonged with extended infusions.

## Data Availability

Not applicable.
